# Approaching sexuality in LGBTQIAP + patients with cancer: scoping review

**DOI:** 10.1186/s12889-023-16170-0

**Published:** 2023-06-30

**Authors:** Tássia Santos Rodrigues, Ricardo Souza Evangelista Sant’Ana, João Paulo Zerbinati, Lucas Nascimento Souza, Anderson Reis de Sousa, Christine Maheu, Simone de Godoy

**Affiliations:** 1 Hospital Sírio-Libanês, São Paulo, Brazil; 2 University of São Paulo at Ribeirão Preto College of Nursing, 3900 - Vila Monte Alegre, São Paulo, Ribeirão Preto 14040-902 Brazil; 3 Faculty of Philosophy, Sciences and Letters at Ribeirão Preto, University of São Paulo, São Paulo, Ribeirão Preto Brazil; 4 Hospital São Rafael, Bahia, Salvador, Brazil; 5 School of Nursing, Federal University of Bahia, Salvador, Bahia, Brazil; 6 Ingram School of Nursing, McGill University, Montréal, QC Canada; 7 University of São Paulo at Ribeirão Preto College of Nursing, São Paulo, Ribeirão Preto Brazil

**Keywords:** Sexuality, Oncology, Sexual and gender minorities, Cancer

## Abstract

**Background:**

When individuals in the SGM group are diagnosed with cancer and undergo treatment, they experience changes in physical, mental, sexual and spiritual dimensions, which can negatively impact sexual desire, as well as satisfaction and sexual health as a whole. This study aims to examine the existing scientific literature on how healthcare professionals approach sexuality in cancer patients who belong to the SGM group. The SGM group is particularly vulnerable, and the challenges they face in terms of psychosocial and emotional health are further exacerbated by the oncological treatment they receive. Therefore, specialized attention and support are necessary to address their unique needs.

**Method:**

To conduct this study, a scoping review was performed following the guidelines established by the Joanna Briggs Institute. By synthesizing the available evidence, this study aims to provide insights and recommendations for healthcare professionals to improve the care and support provided to SGM individuals with cancer. Guiding question: “how do health professionals approach sexuality in cancer patients in a minority group?”. The search was carried out in PubMed, Science Direct, Scopus, Web of Science, Virtual Health Library, Embase databases and Google Scholar in addition. Specific criteria were used for Evidence source selection, Data mapping, assurance, analysis, and presentation.

**Results:**

Fourteen publications were included in this review for the final synthesis, which indicated that the approach to the sexuality of sexual and gender minority groups is based on research whose character is limited in terms of producing care and health care that is congruent in gender and sexuality. The analysis of scientific articles showed that one of the biggest challenges and priorities of health services today is to reduce disparities and promote equity in health for SGM people.

**Conclusions:**

This study reveals a significant gap in addressing the sexuality of SGM groups within cancer care. Inadequate research impedes the provision of consistent and inclusive care for SGM individuals, which has a negative impact on their overall wellbeing. Reducing disparities and promoting healthcare equity for SGM individuals must be a top priority for health services.

## Background

Sexuality encompasses various aspects such as thoughts, desires, attitudes, fantasies, values, behaviours, techniques, roles, and relationships. When individuals in the Sexual and Gender Minority (SGM) group are diagnosed with cancer and undergo treatment, they undergo profound physical, mental, sexual, and spiritual changes. These changes can have a negative effect on sexual desire, sexual satisfaction, and sexual health as a whole. As a result, the oncological diagnosis induces significant changes in the physical, mental, sexual, and spiritual dimensions, resulting in increased psychosocial and emotional vulnerability. Therefore, it is imperative that healthcare providers provide adequate monitoring and support to assist cancer patients in coping with the disease and managing treatment-related complications [1]. They may need to undergo radical surgeries that significantly alter their bodies, leading to a negative self-image and triggering feelings of anguish, stress, and dissatisfaction. These emotional experiences have a direct impact on sexual desire, frequency of sexual intercourse, and the ability to achieve orgasm, generating low self-esteem and diminished sexual satisfaction. Therefore, individuals within SGM groups require specialized sexual health intervention to enhance their overall quality of life [1, 2].

Sexuality is one of the dimensions evaluated in quality-of-life questionnaires, playing an important role in the person’s balance and well-being. Sexuality is influenced by biological, psychological, social, economic, political, cultural, religious, and spiritual factors, which can be modified during the course of life [1]. The World Health Organization (WHO) defines sexuality as a central aspect that involves thought, desire, attitude, fantasy, values, conduct, gender roles, sexual orientation, intimate relationships and/or reproduction [3].

Specifying such concepts, gender is defined as a set of characteristics linked to the personal experience of masculinity and/or femininity. Gender identity consists of how the person subjectively feels and identifies themselves in relation to gender models and may be in line with the gender assigned based on their sex/phenotype at birth (cisgender) or not (transgender) [4].

Sexual orientation concerns the attraction, behaviour, and desire of a person in relation to another in terms of affective-sexual relationships. This relationship can be with people of the same sex (homosexual), of the opposite sex (heterosexual), or both sexes (bisexual). There is also a category called queer, a term referring to people outside gender norms and sexual categories [5].

The term SGM refers to a group of people outside the cis-heteronormative sexual pattern with which most of the population identifies. SGMs suffer discrimination and exclusion, not having access to many basic rights regarding economic, health, education, and other social aspects. Such factors lead to the marginalization and accentuated risk of this population, including in relation to health [6].

The diagnosis and oncological treatment can negatively impact sexual desire, as well as satisfaction and sexual health. Thus, oncological treatment can bring even more fragility to the psychosocial and emotional health of the SGM. The lack of knowledge and prejudice among health professionals can negatively impact the healthcare provided to Lesbian, Gay, Bisexual, Transgender/transsexual, Queer/Questioning, Intersex, Asexual, Pansexual and other individuals (LGBTQIAP +), resulting in difficulties in accessing early diagnosis and possibly delaying cancer treatment. This can lead to a lack of continuity in healthcare for this community, which includes individuals with diverse identities and life experiences that are not explicitly reflected in the abbreviation. One contributing factor that hinders proper follow-up of LGBTQIAP + individuals, who encompass diverse identities beyond the abbreviation, is the omission of questions about their gender identity and sexual orientation in socio-demographic data collection forms. In order to address these obstacles, it is essential that healthcare providers provide specialized and compassionate care that meets the specific needs of the LGBTQIAP + community. This includes ensuring that the entire oncology team provides a differentiated service with humanized and qualified care [7, 8].

The training of healthcare professionals, which focuses primarily on heteronormative populations, frequently influences prejudice. As a result, it lacks awareness of individualized care requirements and fails to account for sexual diversity in the systematization process [7, 8].

Research on SGM with cancer is necessary as these populations face unique challenges and barriers to accessing quality oncology care. These populations may face a lack of understanding and awareness on the part of healthcare professionals, which can lead to delays in diagnosis and treatment, as well as inadequate care. Discrimination and stigma can also have a negative impact on health and treatment outcomes. It is necessary to understand better the specific health needs of these populations and the obstacles they face, as well as to develop more effective interventions and policies to improve cancer care and treatment outcomes [7, 8].

This study sheds light on the importance of cancer care in the context of SGM patients. Cancer patients who identify as LGBTQIAP + have unique healthcare needs that necessitate a humanized and inclusive approach. To accomplish this, healthcare professionals must consistently seek out the best available scientific evidence to comprehend the specific needs associated with sexual and gender diversity. Thus, the objective of this study is to explore the existing scientific literature on how healthcare professionals address the topic of sexuality in SGM cancer patients.

## Method

This study utilizes a scoping review methodology, following the guidelines recommended by the Joanna Briggs Institute (JBI) [9]. The review conforms to the criteria outlined in the Preferred Reporting Items for Systematic Reviews and Meta-Analyses Extension for Scoping Reviews (PRISMA-ScR) Extension for Scoping Reviews [10].

### Protocol and registration

The protocol for this revision is available in the Open Science Framework (OSF), registered with the following Digital Object Identifier (DOI): 10.17605/OSF.IO/ACGBE [11].

### Research question

For the elaboration of the guiding question, the acronym PCC was used—Participants/Concept/Context— [9], assigning, in this order, “P”—oncological patient of the minority group; “C”—approach to the theme "sexuality" in minority groups; “C”—how oncology professionals approach the theme of sexuality in the minority group. Therefore, the question was: “How do health professionals approach sexuality in cancer patients in a minority group?”.

### Search strategy

Initially, a pilot search was performed in the google scholar databases in the months of July and August 2021, aiming to identify which descriptors are the most recurrent in studies related to the theme. Subsequently, adjustments were made to the search strategy, especially in terms of standardization, taking into account the various databases used. This process involved consulting with the librarian from the corresponding author’s institution to ensure accuracy and consistency.

The search was conducted between September and October 2021 by two independent examiners (TSR, CSRS) in the following databases: National Library of Medicine National Institutes of Health (PubMed) (PubMed), Scientific Electronic Library Online (SciELO), Science Direct, Scopus, Web of Science, and Biblioteca Virtual em Saúde (BVS). The main descriptors, keywords and synonyms used were “sexuality” OR “sexual” AND “gender minorities” OR “sexual and gender Minorities” AND “oncology” OR “cancer”.

For this study, in addition, the "snowball" technique allowed the identification of additional sources to the references. This technique was based on the analysis of the reference lists of the articles included in the database searches. After selecting the studies, it was also necessary to perform a new search on academic google to actively locate other studies on the subject in question carried out in different regions of the world.

### Selection criteria

We included studies, regardless of the type of method, which addressed the theme of sexuality of minority sexual groups and gender, available in full in Portuguese, English and Spanish. Studies focusing on sexual behaviors, as well as those related to HPV infections and HIV transmissibility, were excluded, as this was not the focus of this article. There was no restriction of the search in the time frame to cover as many articles as possible.

### Selection of sources of evidence

The located studies were exported to EndNote® Web software to identify and remove duplicates. After that, the data were exported to the Rayyan web application, which allows masking during the selection and screening of studies, favoring reliability and methodological precision. Eligibility was double-assessed by two examiners (TSR, RSE), and, in the end, a third examiner (SG) evaluated the cases in which there was disagreement in the eligibility stage in order to resolve the analysis impasse between the first (TSR) and second (CSR) examiners.

### Data mapping and extraction

After defining the sample, the data was extracted. Seeking to minimize methodological flaws, this stage was carried out by two examiners (TSR, RSE) so that each one carried out the analysis of all included studies, namely: title, country/year of publication, method, and aspects of sexuality of minority sexual groups and gender. Next, the collected information was compared in order to identify, above all, if all aspects of the sexuality of minority sexual groups and gender were located.

### Data analysis and presentation

Data analysis and presentation of results entail providing an overview of the existing literature on the approach to sexuality care for SGM oncological patients. In the final stage, Table 1 is used to group, map, and describe the results in an effort to provide an exhaustive overview of the published material. The focus is specifically on the sexuality care approach for SGM cancer patients. It is important to note that scoping reviews do not involve the analytical synthesis of results [12].

**Table 1:** Summary of the results found

*Author/Year Country*	*Population*	*Objective*	*Method*
Almont et al., 2019 France [13]	All medical, paramedical, or administrative professionals who participated in the 4th Cancer, Sexuality and Fertility Meeting, in Toulouse	To assess attitude, knowledge, communication, and interaction in the clinical practice of health professionals providing oncosexological care	Retrospective cross-sectional study
Cathcart-Rake et al., 2018 USA [14]	Not applicable	To describe cancer treatment for SGM patients in the current literature and to identify knowledge gaps that hinder the understanding of the unique needs of this populations	Integrative review
Cathcart-Rake et al., 2019 USA [15]	504—National Cancer Institute (NCI) Community Oncology Research Program (NCORP) health providers	To report the percentage of NCORP practice regarding data collection about patients with sexual orientation and gender identity (SOGI)	Quantitative study
Curmi et al., 20116 Australia [16]	Nine who self-identified as lesbian women	To provide deeper insights into the experiences of lesbian women in accessing cervical cancer screening and to inform strategies to increase the uptake of these services for this group of women	Qualitative Study
Drysdale et al., 2021 Australia [17]	Not applicable	Examined key findings regarding the feasibility, acceptability and efficacy of evaluated intervention studies conducted in high income settings and published in peer reviewed literature (2014–2020) by combining evidence of both cancer risk-reducing behavioral interventions and screening and preventative practice interventions	Scoping review
Fish et al., 2019 UK [18]	30 LGBTQIA + patients with different types of cancer	To identify which potential salutogenic factors LGBTQIA + cancer patients can recur and how can this be improved in cancer care?	Qualitative study
Griggs et al., 2017 USA [19]	SGM	To assess needs and establish understanding standards for SGM cancer patients and survivor populations, as well as oncology workforce members who identify themselves as members of SGM communities	Quantitative study
Kamen et al., 2019 USA [20]	273 LGBTQIAP + people in the United States	To better understand the experiences of LGBTQIA + cancer patients and their recommendations for cancer healthcare professionals	Qualitative study
Kano et al., 2020 USA [21]	Oncology researcher and provider	To develop a training plan for health professionals to increase health equity in the care of oncological sexual and gender minority (SGM) patients	Quantitative study
Lisy et al., 2018 Australia [22]	Not applicable	To explore the cancer care experiences and unmet needs of people who identify as a sexual or gender minority	Qualitative Systematic Review and Meta‐Synthesis
Margolies et al., 2018 USA [23]	Not applicable	To review the current state of knowledge about LGBTQIA + cancer patients focusing on lack of data, need for a culturally competent healthcare system, and personalized education for LGBTQIA + patients	Integrative review
Radix et al., 2018 USA [24]	Not applicable	To define an overview of the importance of cultural competence in the care for the LGBTQIA + population to provide steps to improve care quality provided by oncology nurses and other health professionals	Literature review
Schabath et al., 2019 USA [25]	450 oncologists from 45 American Medical Physician Masterfile cancer centers	To identify potential attitudinal, knowledge, and institutional practice gaps regarding the LGBTQIA + population	Quantitative study
Shetty et al., 2016. USA [26]	388 oncology health care providers, including all medical doctors (MD), medical assistants (MA), and advanced registered nurse practitioners (ARNP)	To assess the knowledge, attitudinal, and behavioral practices of healthcare providers assigned to LGBTQIA + patient care	Descriptive stratified analyses

## Results

**Fig. 1:**
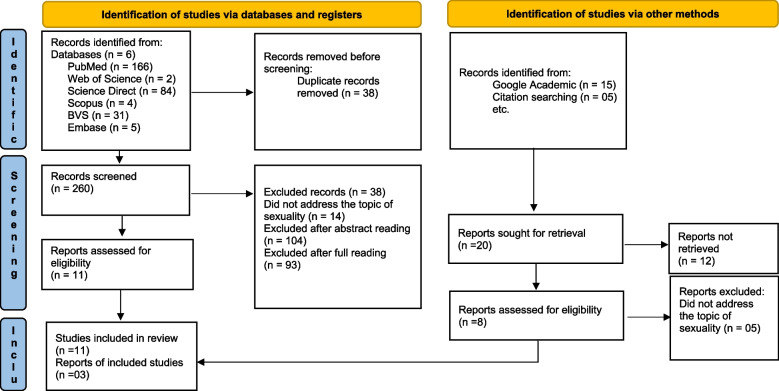
Study selection flow diagram (PRISMA model*) *From: Page, M.J., McKenzie, J.E., Bossuyt, P.M., et al. The PRISMA 2020 statement: an updated guideline for reporting systematic reviews. BMJ 2021, 372(71). https://doi.org/10.1136/bmj.n71. For more information, visit: http://www.prisma-statement

Database searches were completed in early September and repeated in late October 2021. From the initial search, 298 publications were identified. Due to the exclusion and inclusion decision, the final sample of 14 articles was reached for this review. Figure 1 summarizes the search strategy performed for each electronic database and the number of studies selected.

In general, it was noticed from the literature reviewed that significant progress has been made in the development of new cancer treatments, such as oncological drug therapies and multidisciplinary care, which aim to improve patient survival and quality of life. However, it is important to note that this advancement has not been emphasized equally for SGM groups. Compared to the general population, the SGM population faces greater and distinct risk factors and frequently receives insufficient assistance to meet their specific needs. This trend is supported by numerous studies in the scientific literature that indicate a dearth and delay in the development of targeted interventions for SGM groups. To further emphasize this point, Table 2 provides a summary of research-derived recommendations emphasizing the pressing need for targeted actions to address the unique needs of SGM populations.

**Table 2:** Summary of recommendations extracted from research

*Author/Year Country*	*Key Findings*
Almont et al., 2019 France [13]	The need for improvement levels such as oncosexology development in specific educational and practical training programs was evidenced Consolidating information, counseling, and therapeutic education with formal oncosexology procedures implemented for the patient to prevent sexual disorders during cancer treatment and encouraging patients to communicate their sexual difficulties
Cathcart-Rake et al., 2018 USA [14]	***Four humanized and national assistance strategies were suggested to build public policies to fill the gaps found in SGM studies, including:*** Identification of SGM individuals; Staff training in culturally sensitive approaches to SGM cancer patients; Inclusion of messages to support the SGM community in the waiting room; Individualized cancer care
Cathcart-Rake et al., 2019 USA [15]	***Of the 221 practice groups that responded to SOGI questions,*** 14 practice groups (6.3%) collected information on SOGI; 39 practice groups (17.6%) only collected information on sexual orientation; 9 practice groups (4.1%) collected only gender identity information; 159 practice groups (71.9%) collected no information on SOGI
Curmi et al., 2016 Australia [16]	They include providing a friendly, non-judgmental environment for lesbian women when accessing screening services Provide additional education to health professionals on the proper provision of care to lesbian women, without discrimination through heteronormativity Approaches such as referring women or offering them specific health services and/or education for women who self-reveal themselves as lesbians can combat issues associated with stigma by accessing health information Contents related to the health of gays and lesbians could be included in the curricula of undergraduate nurses and other health disciplines. This can help to raise awareness of the need for screening and more sensitive health care for this group of women
Drysdale et al., 2021 Australia [17]	Each has its own unique health needs and presents a significant diversity. In addition, experiences of gender diversity, as well as how gender intersects with other aspects of a person's identity (e.g., sexuality, race, class, ethnicity) Additional attention should be paid to incorporating this diversity into the design process to ensure that all subpopulations are clearly defined and represented through the segmentation process One of the ways to improve community segmentation is through meaningful consultation with relevant communities The value of incorporating peer experience into the design and delivery of the intervention was observed in several of these studies, which is in line with the literature that points to the benefit of members of the affected community being consulted and, ideally, engaged as collaborating partners and co-investigators, in research conducted within LGBTQ communities community-based research on LGBTQ needs and the interventions developed to respond to those needs, along with changes in health professionals' attitudes toward LGBTQ people and understandings of their health risks and intervention needs, are essential for the targeting of cancer prevention and screening interventions to be truly effective
Fish et al., 2019 UK [18]	***Three topics were defined as part of the analysis:*** Authenticity as a driver of disclosure in cancer treatment; Partners as a (potential) salutogenic resource; Creation of safe and curative environments conducive to disclosure Results are reported and discussed regarding three interrelated concepts of the current theory of salutogenesis, including a sense of coherence, resources of generalized resistance, and healing environments that can facilitate sexual orientation disclosure
Griggs et al., 2017 USA [19]	***Five areas of recommendations were outlined to address the needs of both cancer-affected SGM populations and members of the oncology workforce who identify themselves as SGM:*** Patient education and support; Workforce development and diversity; Quality improvement strategies; Policy solutions; Strategic research ***These recommendations are expected to provide greater outreach and educational support for SGM patients:*** Increased SGM cultural competence training for providers; Improved quality of care metrics that include sexual orientation and gender as training variables; Increased data collection for future research addressing the needs of SGM communities
Kamen et al., 2019 USA [20]	Professionals caring for LGBTQIA + cancer patients should: Provide a safe space to welcome them; Ask about and professionally respond to patient identities and identifiers; Include support people to provide relevant care related to the patients’ gender identity and address the effects of treatments on sexuality; All professionals providing direct and indirect care need gender diversity training; Recognizing the strengths of LGBTQIA + cancer patients can improve professional/patient relationships
Kano et al., 2021 USA [21]	***Training plan formats can be static or interactive and can be combined with other strategies for greater learning impact;*** Students can be immersed in small face-to-face workshops for tense skill learning and exposure to content knowledge, and then participate in online webinars and receive further instructions to reinforce skills and knowledge, developing mastery over time Training on SGM cancer issues in a variety of formats will also help students acquire this information. Including the topic as an elective part of healthcare professional curricula proves challenging Research grants are a viable strategy for more intensive one-year or multi-year training when the knowledge of qualified research mentors is necessary to stimulate funding and to reward researchers’ initiatives With the guidance of experienced researchers, trainees can learn how to address gaps related to SGM cancer patients, practicing collecting and interpreting data from members of SGM groups and developing recommendations or improvement interventions, thus strengthening the workforce Organizing multiple trainees into training programs dedicated to cancer topics relevant to SGM groups would be a way to strengthen the workforce more quickly
Lisy et al., 2018 Australia [22]	Avoid assumptions of heterosexuality Avoid heteronormative language and information Enquire about sexual orientation and gender identity in a sensitive and respectful manner If lesbian, gay, and bisexual (LGB) status is disclosed, respond in a positive and reassuring manner Develop competence in discussing sexual matters with LGB people; when needed, refer to other services or seek additional information Include same‐sex partners in care and treat same‐sex partners with respect and courtesy Provide tailored information in response to individual needs, for example, regarding different treatment options or side effects of treatment Where available, recommend appropriate support groups for LGB people and their carers Display LGB/LGBTI images, logos, and other materials Where possible, provide relevant, inclusive supportive resources, including written information, for LGB people with cancer and their carers Include LGB material in cultural competency and diversity training for health care professionals (HCPs) Include LGB sexuality in education for HCPs Link to LGB‐specific or friendly support groups or services, if available Provide and adhere to clear anti‐discrimination policies
Margolies et al., 2018 USA [23]	Most medical record forms do not encourage or allow LGBTQIA + to disclose their sexual orientation and/or gender identity, resulting in lack of research and in dangerous invisibility in LGBTQIA + patient care To date, no national cancer registry collects this information, leaving LGBTQIA + cancer patient care deficient in providing important data. While some nurses and other healthcare professionals bypass forms and directly ask patients about their identities, this information is often unrecorded, not being considered in patient care Many publications come from smaller research with LGBTQIA + patients, either through direct reach or from health information surveys
Radix et al., 2018 USA [24]	LGBTQIA + people who identify their SOGI generate new health promotion behaviors LGBT cultural competence requires dynamics and multilevel systemic change that includes provider education, physical body care, environment, administration acceptance, and inclusion of LGBT community voices through outreach and with a diverse team
Schabath et al., 2019 USA [25]	Health professionals with specific knowledge on understanding LGBTQIA + issues improve the quality of care. As noted in this study, there was great interest in receiving education about LGBTQIA + health needs and greater confidence in the ability of well-trained providers to treat these patients
Shetty et al., 2016. USA [26]	Few physicians felt that they had the necessary skills to address sexual orientation issues, reporting lack of training in undergraduate courses and in medical residency, thus not feeling confident in approaching the issue with their patients. However, they were willing to receive training and education to offer a service that could advise, refer, and educate this population on risk behaviors and specific means of prevention

This study examined a selection of publications focusing on the sexuality of cancer patients from minority groups, as this topic has been understudied, as evidenced by the conducted mapping. The majority of the publications analyzed originate in North America, with a smaller proportion coming from Europe. Some studies published in the previous decade were excluded because their full versions were unavailable. Notably, this review represents a subset of the subject's larger body of literature. Despite these limitations, the findings of this study contribute to our understanding of the current state of research on sexuality in minority cancer patients.

## Discussion

The literature mapping revealed the landscape of cancer care focused on the health of SGM groups. In the following sections, we discuss the findings based on thematic categories of analysis.

### Research on sexual and gender minorities with cancer

The analysis of scientific articles revealed that one of the biggest challenges and priorities of health services today is to reduce disparities and to promote health equity for SGM people. Prevention, screening, treatment acceptance, and future care are daily barriers and the main causes of health inequality in SGM, justified by a lack of training and technical-scientific preparation of health professionals, who have limited capacity to care for this population. Another cause is the lack of scientific funding directed to this group [15, 20].

These issues contribute to the limitations in addressing sexuality with SGM cancer patients. Thus, organizational institutions should endeavor to reduce this disparity, ensuring that the SGM population receives tailored, high-quality care [19, 21].

International oncology organizations have discussed the need for professional and institutional qualification and the creation of strategies to expand healthcare access and improve cancer screening in these groups, promoting an inclusive and discrimination-free environment for the LGBTQIAP + community [14, 15, 23].

International institutions define SGM as lesbian, gay, bisexual, transgender, queer, and asexual people, as well as those with a medical sexual development disorder who identify as intersex. Due to the lack of effective screening, the SGM population may present more advanced stages of the disease at the time of diagnosis due to the lack of health insurance coverage or campaigns directed mainly to the heterosexual group. However, discrimination and refusal of care are the main factors reported that justify screening ineffectiveness, leaving this population unassisted [14, 16, 22, 23].

### Impact of cancer treatment on the lives of people from SGM groups

The impact of cancer treatment on the lives of people belonging to SGM groups can be significant and may differ from the experiences of cisgender and heterosexual individuals. People in SGM groups may face unique challenges and barriers to accessing cancer care, including discrimination and stigma by healthcare providers and a lack of cultural competence in cancer treatment.

In the United States of America (USA), the scientific community identified significant differences in the prevalence of diseases and risk factors for the LGBTQIAP + population compared to their heterosexual peers. SGM cancer patients have specific psychosocial challenges, such as stress, anxiety, depression, and suicide, and thus may have a worse response to treatment [14, 15, 19, 21, 23].

In the USA, women have a higher rate of obesity and an increased risk of developing breast cancer. This association has not been studied in the SGM group. Still, some studies bring other factors that influence the development of cancer, including reduced pregnancy rate, smoking, and use or lack of certain hormones. Gay men have a higher risk of anal cancer because its main cause is the Human Papillomavirus (HPV), a sexually transmitted infection. Transsexual women can have testicular or prostate cancer [14, 16, 17, 21–23].

Compared with heterosexuals, SGM people of both sexes and genders are more prone to drug and alcohol abuse and smoking due to their history of rejection, homophobia, and social exclusion from the community in which they live, this phenomenon is called minority stress (MS). Due to these external factors, this group has a higher risk of developing certain types of cancer than the general population [14, 19, 23].

Cancer treatment can leave long-term sequelae that sometimes can last for years, such as asthenia, pain, stress, nausea, alopecia, loss of part of an organ, and early menopause (loss of libido, vaginal dryness, hot flashes). Such consequences can lead to low self-esteem, loss of functionality, and decreased quality of life [16–18].

Cancer treatment can impact sexuality and sexual function, causing sexual dysfunction regardless of gender and sexual orientation. Therefore, a differentiated approach is necessary according to the specificity and individuality of each group and patient. Sexual dysfunction is defined by the American Psychiatric Association as a change in one of the phases of the sexual cycle: desire, arousal, orgasm, and resolution [14, 15, 19, 21, 23].

The literature demonstrates the need to include cultural competence training that covers LGBTQIAP + sexuality, relationships and other LGBTQIAP + -specific issues in the education of health professionals and to provide clear anti-discrimination policies for health professionals. This is an important issue based on the detection of some health professionals who considered the discussion on LGBTQIAP + sexuality "embarrassing" and described prejudiced behavior towards LGBTQIAP + patients, which could compromise the quality of care and care [16, 22].

There are additional benefits of an intervention geared towards the needs of LGBTQIAP + people, carried out by competent professionals or in safe environments, ensuring that care is appropriate and non-discriminatory [17].

### Approach to sexuality for SGM people

The approach to sexuality for people of sexual and gender minorities should be of respect, inclusion, and support. It is important to recognize that sexual orientation, gender identity, and expression are integral aspects of a person's identity and that everyone has the right to express themselves and their sexuality in an authentic and satisfying way.

“Sexuality” is a broad term encompassing several intrinsic and extrinsic elements, hardly fitting into a single definition. It can be understood as a basic human need that should not be separated from other aspects of life and is not just synonymous with coitus or restricted to orgasm. It is related to energy and experience exchange and physical contact and intimacy, being expressed in feelings, movements, and exchanges [19, 24].

Human sexuality comprises characteristics such as pleasure, reproduction, friendship, love, affection, sexual practices, sexual orientation, and gender. It involves pleasurable tactile sensations, affection, and love arising from marital, fraternal, or friendly relationships. Sexuality is expressed according to historical, sociocultural, familial, and subjective contexts [19, 25, 26].

The main gap to be filled when discussing sexuality with patients is to have a clear and welcoming approach to deconstruct the obstacles inherent in the topic. This study could not identify specific validated instruments to support and facilitate communication and the approach to the sexuality of LGBTQIAP + cancer patients [13].

However, some models, such as the PLISSIT and BETTER help educational interventions provide well-developed and structured approaches, often presenting limited active behaviour change components to support effective implementation in practice.

The BETTER model was structured to help professionals start a conversation about sexuality. The acronym relates to the words: Bring up the subject, the first and main step; Explain*,* when the professional should introduce the subject “sexuality” and show the patient that they are concerned about their quality of life; Tell, which gives the individual the opportunity to expose their concerns and, even if the professional does not know how to answer all the questions, they will find appropriate sources to clarify them; Timing, when everything has its time and the patient will be helped at any stage of the disease and at any time; Educate the patient about the possible effects of cancer treatment; and Record all information in the patient’s medical record [27].

The PLISSIT model, proposed by Jack S. Annon, was also found in the searches and involves a combination of four elements: Permission, asking for the patient’s permission to talk about sexuality; LI, for Limited Information, as it is often a sensitive topic, it is necessary to limit information in the first contact; SS, for Specific Suggestion, give information to patients as they mention some sexual dysfunction related to the treatment; T, for intensive Therapy, referral to a specialist if there is no condition or competence to solve any identified dysfunction [28].

Both models have shown a positive effect in terms of patient-professional communication and approximation, regardless of their level of knowledge on sexuality issues. Communication skills and the search for practices based on high-level scientific evidence related to psychosexual problems are necessary, in addition to investment in new studies to demonstrate the effectiveness of models to approach sexuality with LGBTQIAP + cancer patients, contributing to the establishment of a relationship of trust between the health professional and the patient.

The female and male versions of the Sexual Quotient Scale (QS-F and QS-M) were developed and validated in Portuguese to evaluate the sexual response considering six spheres: sexual desire, sexual arousal, vaginal lubrication, orgasm, sexual satisfaction, and pain. It is an easy-to-understand tool to diagnose sexual dysfunction. The collected data create a specific care plan to improve the patient’s sexual performance. These questionnaires can be used for LGBTQIAP + people with cancer [29].

As for professional competencies, the oncology nurse would be a representative figure to work on sexuality issues in several dimensions, especially considering the holistic training of this professional, which facilitates a conversation about the subjective issues that the topic requires. In addition, it is a profession that anchors the work process in theories based on scientific knowledge, demonstrating a tendency to look at the health-disease process in a biopsychosocial way. However, nurses need to learn about the topic through better scientific evidence, training courses, and seminars, among others [30].

In this sense, it is important to emphasize that the profession follows the North American Nursing Diagnosis Association (NAND-I) guidelines, a professional nursing organization to standardize nursing terminology. This organization defines sexual dysfunction as an unsatisfactory or inadequate sexual function response during arousal and/or orgasm [31].

Care systematization after raising and clinically analyzing the problem based on domain eight on sexuality, class 1 – sexual identity, and class 2 – sexual function, shows that sexuality can be traced under a line of individual care directed to the patient’s best care. Thus, some studies are conducted to analyze strategies for better-approaching sexuality with SGM people, creating an educational plan for professionals directly linked to the patient [31].

Some of the included studies also showed factors negatively influencing more inclusive care for the LGBTQIAP + community with cancer, such as lack of professional training and knowledge on specific health problems and explicit (conscious) and implicit (unconscious) prejudicial attitudes, resulting in inadequate care for the SGM group [14, 19, 23].

The American Society of Clinical Oncology (ASCO) recommends the development of strategies in the field of patient education and support, workforce development and diversity, health quality improvement, policy solutions, and new research formats not only in patient care, but including the family and/or support network and oncology workers who identify themselves as members of SGM, providing a high-quality health care environment with safety and empathy. Measures to improve the role of the patient’s navigator can be useful, with professionals directing and encouraging the search for referenced support networks to avoid cases of homophobia or embarrassment [26].

This perspective shows the importance of creating educational strategies, including information on prevention, screening, most common illnesses, treatments, and side effects that can affect the well-being and quality of life of the LGBTQIAP + community. In addition, some important measures to provide a safe health service environment for this community include the creation of waiting rooms and individual appointments; groups, programs, safe spaces, and rehabilitation for patients and families; and welcoming environments with appropriate facilities to provide safety during appointments and in the waiting room, such as gender-neutral/inclusive private bathrooms and offices [14, 19, 24].

The authors suggest that SGM patients receive competent and effective services that prioritize diversity. This includes developing self-evaluation skills, managing the dynamics of differences, acquiring institutional cultural knowledge, and adapting to the cultural diversity of individuals and communities. It is essential, however, for professionals to receive training in order to address prejudices and taboos rooted in societal and historical values [14, 32].

Discrimination against the patients’ sexual orientation and/or gender identity influences the health/disease process, aggravating the suffering resulting from prejudice and social stigma. Health professionals must promote comprehensive health, eliminating discrimination and institutional and social prejudice, thus reducing inequalities [19, 24, 27].

Therefore, our findings showed it is urgent to develop and strengthen research on specific care for each type of cancer in the LGBTQIAP + community and on the impact of the disease on the sexuality and quality of life of these patients. As research is strengthened in this scenario, individualized training based on the best scientific evidence becomes possible for the entire team providing care to community members. Training should raise the awareness of professionals about the relevance and magnitude of the topic, in addition to demonstrating the negative impacts of discrimination and prejudice on the LGBTQIAP + community and how to deal with these issues at all stages of cancer diagnosis and treatment.

The production of health care for the LGBTQIAP + population with cancer must be extended to all levels of health care, such as primary Health care, where early cancer diagnosis can occur. Integrality in health aimed at this key population will require essential components from health professionals for a welcoming and judgment-free practice, such as the exercise of empathy, advocacy, the maintenance of human rights and non-discrimination, in any form or expression of sexual orientation or gender identity [33].

Therefore, it will be necessary to protect key populations from vulnerabilities and social inequities in health as a way of making them less susceptible to facing other difficulties in maintaining their treatments, guaranteeing psychosocial well-being and quality of life. In times of pandemics COVID-19 and other health crises such as the advent of Monkeypox, it is essential that health care be guided by ethics, reducing stigma, prejudice and discrimination in the health of the LGBTQIAP + population, how to minimize the impacts on physical and mental health and spirituality of this population [34, 35].

The findings presented in this review can serve as a valuable basis for making informed healthcare decisions. They can inform the evaluation of LGBTQIAP + specific care models, service management, and healthcare provision. In addition, these findings can encourage the implementation of specific actions designed to support cancer patients, their families, and the entire healthcare team.

## Conclusions

It is pertinent to address the issue that this scoping review predominantly revealed works from the US, France, and the UK. This bias may be attributed to the concentration of top publishers in these countries, inadvertently influencing the results of scoping assessments. Consequently, this scoping review acknowledges this significant limitation in terms of the results obtained. It is essential to recognize the relevance of this limitation to prompt future research to adopt methodological steps that actively seek contributions from other continents and regions and, thereby, addressing and rectifying the North American and European bias. To encourage the development of new research and drive practice and change in public policy, we advocate for the inclusion of questions about sexual and gender identity in population-level surveys, including cancer registries and all healthcare settings. Such initiatives are likely to enhance our understanding of LGBTQIAP + individuals’ patient-reported outcomes and facilitate improvements in their care.

The present research showed scarce literature on approaching sexuality with cancer patients, which was even more scarce when the search was restricted to the LGBTQIAP + community. Therefore, there is an urgent need to increase human, material, and financial resources for research/teaching in this scenario, as well as the development of effective professional training strategies. It is also necessary to work hard to raise the awareness of institutions providing cancer care about the importance of creating an inclusive environment to better welcome patients.

Therefore, we must strengthen research on screening, estimation, epidemiology, triage, diagnosis, treatment, survival, and palliative care for all members of the LGBTQIAP + community with cancer, approaching sexuality during the entire patient follow-up. These data show the possibility of outlining individualized, inclusive, and welcoming care, assuring the right to access health actions and services early and promoting the health of LGBTQIAP + patients who need specific care.

The provision of cancer care for SGM patients presents difficulties for healthcare professionals due to societal and cultural influences, limited scientific knowledge, and inadequate training in gender and sexuality. To address these obstacles, educational institutions must include this topic in the curriculum of all healthcare professionals, ensuring that future practitioners are equipped with the necessary sensitivity and understanding. By fostering an inclusive approach to care that is devoid of discrimination and prejudice, we can create a healthcare setting that is increasingly conducive to the health and quality of life of SGM patients.

## Data Availability

Not applicable.

## Abbreviations

ASCO: American Society of Clinical Oncology
BVS: Biblioteca Virtual em Saúde
COVID-19: Coronavirus Disease 2019
GS-F: Sexual Quotient Scale – Female Version
GS-M: Sexual Quotient Scale – Male Version
HPV: Human Papillomavirus
JBI: Joanna Briggs Institute
LGBTQIAP +: Lesbian, Gay, Bisexual, Transgender/transsexual, Queer/Questioning, Intersex, Asexual, Pansexual and other identities and experiences that are not specifically represented in the abbreviation
MS: Minority Stress
NAND-I: American Nursing Diagnosis Association
OSF: Center for Open Science
PCC: Population, Concept, and Context
PubMed: Library of Medicine National Institutes of Health
SciELO: Scientific Electronic Library Online
SGM: Sexual and Gender Minority
UK: United Kingdom
USA: United States of America
WHO: World Health Organization
